# Zirconoarylation of alkynes through *p*-chloranil-promoted reductive elimination of arylzirconates

**DOI:** 10.3762/bjoc.10.48

**Published:** 2014-02-28

**Authors:** Xiaoyu Yan, Chao Chen, Chanjuan Xi

**Affiliations:** 1 Key Laboratory of Bioorganic Phosphorus Chemistry & Chemical Biology (Ministry of Education), Department of Chemistry, Tsinghua University, Beijing 100084, China; 2 State Key Laboratory of Elemento-Organic Chemistry, Nankai University, Tianjin 300071, China

**Keywords:** alkyne, multicomponent, reductive elimination, zirconate, zirconoarylation

## Abstract

A novel method for the zirconoarylation of alkynes was developed. TCQ-promoted reductive elimination of arylzirconate [LiCp_2_ZrAr(RC≡CR)], which was prepared by the reaction of zirconocene–alkyne complexes with aryllithium compounds, afforded trisubstituted alkenylzirconocenes. This reaction can afford multi-substituted olefins with high stereoselectivity.

## Introduction

**Scheme 1:**
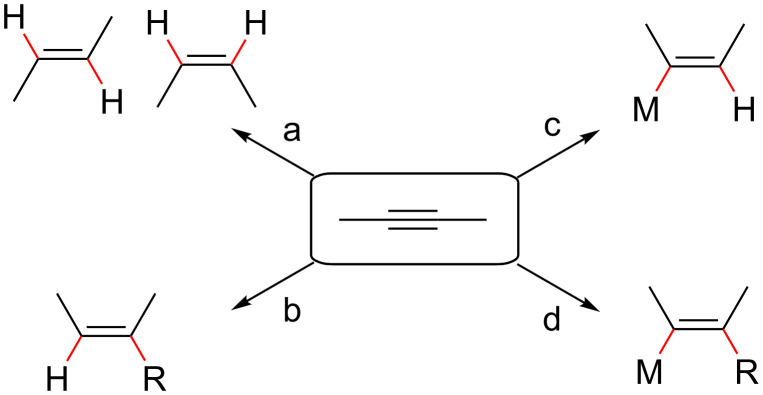
Transformation of alkynes to olefins.

The controlled synthesis of multi-substituted olefins is one of the most challenging tasks in organic synthesis [1–2]. A series of reactions have been developed for the construction of substituted olefins. Among them, an important route is the addition of various reagents to nonactivated alkynes to form substituted olefins. For example, semihydrogenation of internal alkynes can afford disubstituted olefins (Scheme 1, route a) [3–5]. Hydrocarbonation [6–7] and hydrometalation/functionalization [8–11] of internal alkynes can afford trisubstituted olefins (Scheme 1, routes b and c), respectively. The most exciting progress was the carbometalation of internal alkynes [12–23], which involves simultaneous addition of a metal atom and an organic residue to alkynes. The newly formed carbon–metal bond can be used for further synthetic transformation toward multi-substituted olefins [24–36] (Scheme 1, route d).

**Scheme 2:**
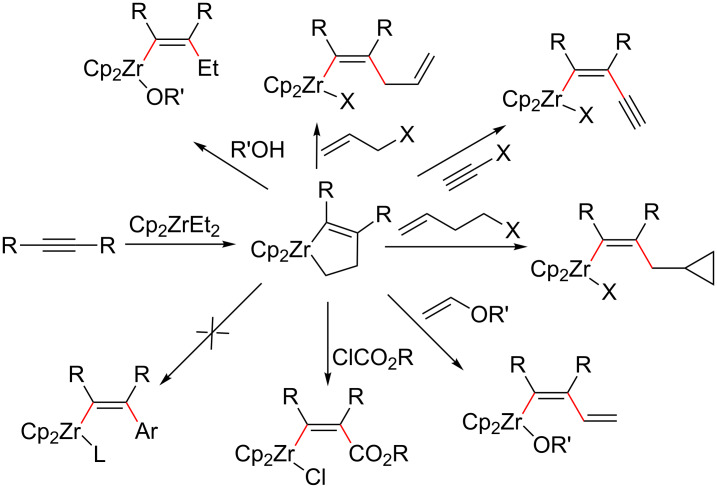
Carbozirconation of alkynes via zirconacyclopentenes.

A large number of carbometalation reactions of alkynes have been reported. Most of the carbometal reagents, which were used, contained Li, Mg, Cu, Zn, B, or Al [12–16]. In the last several decades also zirconium-mediated or -catalyzed organic reactions have been extensively investigated [37–39]. In addition, a series of organic reactions using zirconocene species have been reported, in particular for the reductive coupling of alkenes or alkynes with other unsaturated compounds [40–43]. On the other hand, carbozirconation of alkynes via reaction of zirconacyclopentenes with alcohols, allyl ethers, homoallyl bromides, vinyl ethers, alkynyl halides, and chloroformates gave ethylzirconation [17], allylzirconation [18–19], cyclopropylmethylzirconation [20], vinylzirconation [21], alkynylzirconation [22], and zirconoestification [23] products of alkynes, respectively. However, to the best of our knowledge, this method failed to fulfill arylzirconation of alkynes (Scheme 2).

**Scheme 3:**
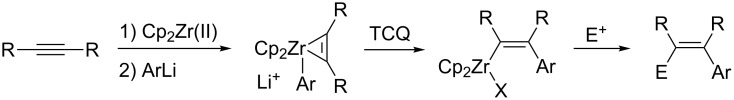
TCQ-promoted reductive elimination of arylzirconate.

Recently, we have reported a *p*-chloranil (TCQ)-promoted reductive elimination reaction of the zirconate complex Li[Cp_2_Zr(C≡CR)_3_] toward geminal enediynes [44]. As part of our ongoing project on organozirconate chemistry [45–48], we envisioned that the use of an aryl ligand instead of one of the alkynyl ligands would provide an arylzirconation product of the alkyne. Herein we describe the TCQ-promoted reductive elimination of arylzirconate to afford an arylzirconation product of the alkyne, which can be converted to multi-substituted olefins through coupling with electrophiles (Scheme 3).

## Results and Discussion

**Scheme 4:**
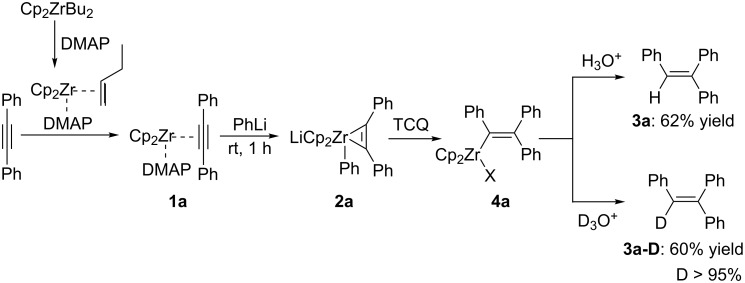
TCQ-promoted arylzirconation of diphenylacetylene.

Initially, the reaction of Cp_2_Zr(PhC≡CPh)(DMAP) (**1a**), prepared by the reaction of Cp_2_ZrBu_2_ [48] with DMAP and diphenylacetylene according to reported literature [49], with phenyllithium produced arylzirconate **2a**. To this mixture, 2 equivalents of *p*-chloranil (TCQ) were added and the reaction mixture was stirred for 12 h at room temperature. After being quenched with HCl solution, the desired triphenylethylene (**3a**) was isolated in 62% yield. When the reaction mixture was quenched with DCl solution, the deuterated product **3a-D** was isolated in 60% yield with >95% of deuterium incorporation (Scheme 4). The deuterium experiment revealed the formation of triphenylvinylzirconocene **4a** as intermediate.

Under similar reaction conditions, a study on the substrate scope was carried out, and the results are summarized in Table 1. When diphenylacetylene was used as starting material, different aryllithium compounds were employed to afford triarylethylene in 38% to 62% isolated yields after being quenched with HCl (Table 1, entries 1–4). Bromination of the reaction mixture by NBS instead hydrolysis afforded bromotriphenylethylene in 37% isolated yield (Table 1, entry 5). When allyl bromide was employed as electrophile in the presence of CuCl, the allyltriarylethylenes were formed in 43% to 45% yields (Table 1, entries 6 and 7). Cross coupling with iodobenzene in the presence of CuCl/Pd(PPh_3_)_4_ afforded tetraarylethylene in 31% yield (Table 1, entry 8). When other diarylacetylene was employed in this reaction, the corresponding products were formed in 36% to 59% yields (Table 1, entries 9–12). No desired product was observed when alkylacetylenes were used.

**Table 1:** Formation of multi-substituted olefins via the reaction of alkynes with [Cp_2_Zr(1-butene)(DMAP)] and aryllithium in the presence of TCQ^a^.

entry	alkynes	aryllithium	electrophiles	products	yield (%)^b^

1	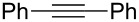	PhLi	HCl	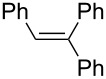 **3a**	62
2	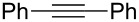	TolLi	HCl	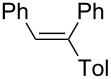 **3b**	58
3	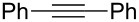	2-ThLi	HCl	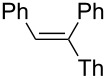 **3c**	47
4	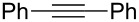	NaphLi	HCl	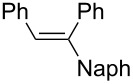 **3d**	38
5	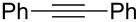	PhLi	NBS	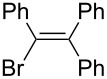 **3e**	37
6	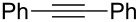	PhLi	allyl-Br	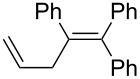 **3f**	45
7	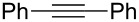	TolLi	allyl-Br	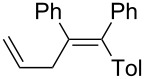 **3g**	43
8	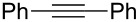	TolLi	PhI	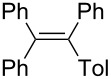 **3h**	31
9	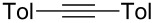	PhLi	HCl	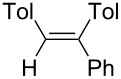 **3i**	57
10	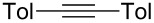	PhLi	NBS	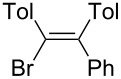 **3j**	36
11	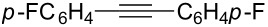	PhLi	HCl	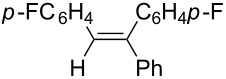 **3k**	59
12	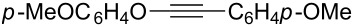	PhLi	HCl	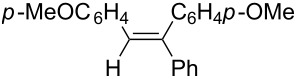 **3l**	52

^a^Reaction conditions: Alkyne (1 mmol), [Cp_2_Zr(1-butene)(DMAP)] (1 mmol), ArLi (2 mmol), TCQ (2 mmol), electrophile (2 mmol). Tol = *p*-tolyl, Th = 2-thienyl, Naph = 1-naphthyl. ^b^Isolated yield.

**Scheme 5:**
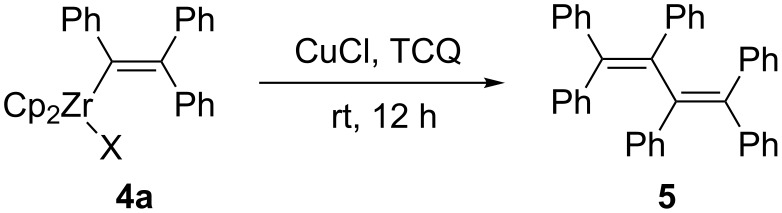
Oxidative dimerization of **4a**.

Recently, oxidative dimerization of alkenylcopper was reported to afford conjugated dienes and polyenes [44,50–57]. When triphenylvinylzirconocene **4a** was reacted in the presence of CuCl and TCQ, the 1,1,2,3,4,4-hexaphenyl-1,3-butadiene (**5**) was formed in 43% isolated yield (Scheme 5). It is noteworthy that in this reaction two molecular alkynes and two aryllithium compounds were coupled in one-pot in the presence of Cp_2_Zr species to afford highly substituted 1,3-butadienes.

**Scheme 6:**
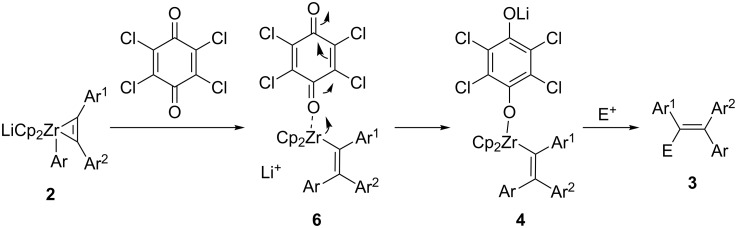
Possible reaction mechanism.

The pathway of the oxidation of zirconate **2** to vinylzirconocene **4** is not yet clear. A possible mechanism is proposed in Scheme 6. Coordination of TCQ to zirconium results in reductive elimination to afford vinylzirconocene(II) **6**. Then intermediate **6** reacts with TCQ to form vinylzirconocene(IV) **4**. The intermediate **4** reacts with electrophiles to afford multi-substituted olefin **3**.

## Conclusion

We have developed a novel method for the zirconoarylation of alkynes through TCQ-promoted reductive elimination of arylzirconate. This reaction can afford multi-substituted olefins with stereoselectivity.

## Experimental

**General Comments**. All manipulations were conducted in Schlenk tubes and under a nitrogen atmosphere with a slightly positive pressure. Unless otherwise noted, all starting materials were commercially available and were used without further purification. Tetrahydrofuran (THF) was refluxed and freshly distilled from dark purple solutions of sodium and benzophenone under nitrogen atmosphere. ^1^H NMR and ^13^C NMR spectra were recorded on 300 MHz and 400 MHz NMR spectrometers with TMS as internal standard. GC–MS spectra were recorded on a Hewlett Packard GC–MS system.

### Typical procedure for TCQ-promoted arylzirconation of alkynes

To a solution of Cp_2_ZrCl_2_ (1.2 mmol, 351 mg) in 5 mL THF, *n*-BuLi (2.4 mmol, 1.5 mL, 1.6 M in hexane) was added at −78 °C and the mixture was stirred for 1 h at the same temperature. To this solution, 4-dimethylaminopyridine (DMAP, 122 mg, 1.0 mmol) was added. The resulting mixture was warmed to room temperature and stirred for 1 h. Diphenylacetylene (1.0 mmol, 178 mg) was added and the mixture was stirred for 1 h at the same temperature. Subsequently, PhLi (2.0 mmol) was added and the solution was stirred for 12 h at room temperature. Then TCQ (2.0 mmol) was added and stirred for another 12 h to afford alkenylzirconocene **4a**. The mixture was quenched with HCl solution to afford product **3a** in 62% isolated yield.

1,1,2-Triphenylethene (**3a**) [58]: ^1^H NMR (300 MHz, CDCl_3_, Me_4_Si) δ 6.93 (s, 1H), 6.99–7.30 (m, 15H); ^13^C NMR (75 MHz, CDCl_3_, Me_4_Si) δ 126.6, 126.8, 127.5, 127.6, 127.7, 128.1, 128.3, 128.7, 129.7, 130.5, 137.5, 140.5, 142.7, 143.5; GC–MS *m*/*z*: 256.

2-Deuterium-1,1,2-triphenylethene (**3a-D**) [59]: The reaction mixture containing **4a** was quenched with DCl, and **3a-D** was isolated in 60% yield. ^1^H NMR (300 MHz, CDCl_3_, Me_4_Si) δ 7.00–7.51 (m, 15H); ^13^C NMR (75 MHz, CDCl_3_, Me_4_Si) δ 127.0, 127.6, 127.7, 127.7 (t, *J*_DC_ = 7.2 Hz), 127.8, 128.2, 128.4, 128.8, 129.7, 130.6, 137.5, 140.5, 142.7, 143.6; GC–MS *m*/*z*: 257.

1,2-Diphenyl-1-(*p*-tolyl)ethene (**3b**) [58]: The reaction was using *p*-tolyllithium instead of phenyllithium, and **3b** was isolated in 58% yield. ^1^H NMR (300 MHz, CDCl_3_, Me_4_Si) δ 2.45 (s, 3H), 7.06 (s 1H), 7.20–7.42 (m, 14H); ^13^C NMR (75 MHz, CDCl_3_, Me_4_Si) δ 21.4, 126.8, 127.6, 127.7, 128.2, 128.8, 129.1, 129.7, 130.6, 137.5, 137.7, 140.7, 140.9, 142.8; GC–MS *m*/*z*: 270.

(*E*)-1,2-Diphenyl-1-(2-thienyl)ethene (**3c**): The reaction was performed using 2-thienyllithium instead of phenyllithium, and **3c** was isolated in 47% yield. ^1^H NMR (300 MHz, CDCl_3_, Me_4_Si) δ 6.69–6.71 (dd, *J*_HH_ = 3.8, 1.1 Hz, 1H), 6.87–6.90 (m, 1H), 6.93–6.96 (m, 2H), 7.04–7.09 (m, 4H), 7.13–7.15 (dd, *J*_HH_ = 4.8, 2.9 Hz, 1H), 7.26–7.36 (m, 5H); ^13^C NMR (75 MHz, CDCl_3_, Me_4_Si) δ 124.8, 126.2, 126.4, 126.9, 127.5, 127.9, 128.1, 128.9, 129.5, 130.0, 136.3, 136.7, 139.5, 148.0; GC–MS *m*/*z*: 262; HRMS: calcd for C_18_H_14_S, 262.0816; found, 262.0813.

(*E*)-1-(1-Naphthyl)-1,2-diphenylethene (**3d**) [59]: The reaction was performed using 1-naphthyllithium instead of phenyllithium, and **3d** was isolated in 38% yield. ^1^H NMR (300 MHz, CDCl_3_, Me_4_Si) δ 6.85 (s, 1H), 7.23–8.17 (m, 17H); ^13^C NMR (75 MHz, CDCl_3_, Me_4_Si) δ 125.4, 125.8, 126.1, 126.3, 127.1, 127.4, 127.5, 128.0, 128.2, 128.4, 128.5, 129.6, 129.9, 131.8, 132.0, 134.1, 137.5, 141.1, 141.4, 142.3; GC–MS *m*/*z*: 306.

1-Bromo-1,2,2-triphenylethene (**3e**) [60]: The reaction mixture containing **4a** was further treated with NBS (2 mmol) for 4 h at room temperature and **3e** was isolated in 37% yield. ^1^H NMR (300 MHz, CDCl_3_, Me_4_Si) δ 6.93–6.97 (m, 3H), 7.02–7.05 (m, 3H), 7.11–7.17 (m, 3H), 7.27–7.33 (m, 3H), 7.35–7.37 (m, 3H); ^13^C NMR (75 MHz, CDCl_3_, Me_4_Si) δ 122.3, 127.1, 127.7, 128.0, 128.1, 128.2, 128.4, 129.7, 130.4, 130.4, 141.1, 141.2, 143.7, 143.9; GC–MS *m*/*z*: 334, 336.

1,1,2-Triphenylpenta-1,4-diene (**3f**) [27]: To the reaction mixture containing **4a**, CuCl (1 mmol) and allyl bromide (2 mmol) were added. The reaction mixture was stirred for 12 h at room temperature and **3f** was isolated in 45% yield. ^1^H NMR (300 MHz, CDCl_3_, Me_4_Si) δ 3.28–3.30 (d, *J_HH_* = 6.1 Hz, 2H), 4.97–5.05 (m, 2H), 5.73–5.79 (m, 1H), 6.91–7.40 (m, 15H); ^13^C NMR (75 MHz, CDCl_3_, Me_4_Si) δ 40.6, 116.1, 126.2, 126.5, 127.1, 127.6, 127.7, 128.0, 128.3, 129.8, 130.0, 131.0, 136.5, 138.0, 140.9, 142.4, 143.1, 143.4; GC–MS *m*/*z*: 296.

(*E*)-1,2-Diphenyl-1-(*p*-tolyl)penta-1,4-diene (**3g**): The reaction was performed using *p*-tolyllithium instead of phenyllithium. After treatment with TCQ for 12 h, CuCl (1 mmol) and allyl bromide (2 mmol) was added. The reaction mixture was stirred for 12 h at room temperature and **3g** was isolated in 43% yield. ^1^H NMR (300 MHz, CDCl_3_, Me_4_Si) δ 2.44 (s, 3H), 3.36–3.38 (d, *J_HH_* = 6.1 Hz, 2H), 5.80–5.89 (m, 2H), 7.01–7.25 (m, 14H); ^13^C NMR (75 MHz, CDCl_3_, Me_4_Si) δ 21.4, 40.6, 116.0, 126.1, 126.3, 127.6, 128.0, 128.9, 129.7, 130.0, 131.0, 136.6, 136.6, 137.7, 140.5, 140.8, 142.5, 143.3; GC–MS *m*/*z*: 310; HRMS: calcd for C_24_H_22_, 310.1722; found, 310.1724.

1,2,2-Triphenyl-1-(*p*-tolyl)ethene (**3h**) [61]: The reaction was performed using *p*-tolyllithium instead of phenyllithium. To the reaction mixture, CuCl (1 mmol), Pd(PPh_3_)_4_ (0.05 mmol), and iodobenzene (2 mmol) were added. The reaction mixture was stirred for 12 h at room temperature and **3h** was isolated in 31% yield. ^1^H NMR (300 MHz, CDCl_3_, Me_4_Si) δ 2.28 (s, 3H), 3.94–7.12 (m, 19H); ^13^C NMR (75 MHz, CDCl_3_, Me_4_Si) δ 21.3, 126.4, 126.4, 127.7, 127.8, 128.5, 131.3, 131.4, 131.5, 136.1 ,140.6, 140.9 ,141.0, 144.1; GC–MS *m*/*z*: 346.

(*Z*)-1-Phenyl-1,2-di(*p*-tolyl)ethene (**3i**) [58]: The reaction was performed using ditolylacetylene instead of diphenylacetylene, and **3i** was isolated in 57% yield. ^1^H NMR (300 MHz, CDCl_3_, Me_4_Si) δ 2.31 (s, 3H), 2.43 (s, 3H), 6.96 (s, 1H), 7.00–7.36 (m, 13H); ^13^C NMR (75 MHz, CDCl_3_, Me_4_Si) δ 21.3, 21.5, 127.4, 127.7, 128.0, 128.3, 128.8, 129.5, 129.6, 130.4, 134.8, 136.6, 137.1, 137.7, 141.8, 144.0; GC–MS *m*/*z*: 284.

(*E*)-1-Bromo-2-phenyl-1,2-di(*p*-tolyl)ethene (**3j**): The reaction was performed using ditolylacetylene instead of diphenylacetylene. After bromination by NBS (2 mmol), **3j** was isolated in 36% yield. ^1^H NMR (300 MHz, CDCl_3_, Me_4_Si) δ 2.18 (s, 3H), 2.25 (s, 3H), 6.80–7.38 (m, 13H); ^13^C NMR (75 MHz, CDCl_3_, Me_4_Si) δ 21.3, 21.4, 122.1, 127.5, 128.2, 128.7, 128.8, 129.6, 130.3, 130.3, 136.7, 137.8, 138.4, 138.5, 143.0, 144.3; GC-MS: 362, 364; HRMS: calcd for C_22_H_19_Br, 362.0670; found, 362.0674.

(*Z*)-1-Phenyl-1,2-di(*p*-fluorophenyl)ethene (**3k**) [59]: The reaction was performed using 4,4′-difluorodiphenylacetylene instead of diphenylacetylene, and **3k** was formed in 59% yield. ^1^H NMR (400 MHz, CDCl_3_, Me_4_Si) δ 6.87–6.78 (m, 2H), 6.90 (s, 1H), 7.05–6.95 (m, 4H), 7.18–7.10 (m, 2H), 7.25–7.35 (m, 5H); ^13^C NMR (100 MHz, CDCl_3_, Me_4_Si) δ115.2 (d, *J* = 21.4 Hz), 115.9 (d, *J* = 21.5 Hz), 127.4, 127.7, 127.9, 128.4, 131.2 (d, *J* = 7.8 Hz), 132.2 (d, *J* = 7.8 Hz), 133.4, 136.1, 141.5, 143.2, 161.6 (d, *J* = 247.4 Hz), 162.3 (d, *J* = 246.7.8 Hz); GC–MS *m*/*z*: 292.

(*Z*)-1-Phenyl-1,2-di(*p*-methoxylphenyl)ethene (**3l**) [59]: The reaction was performed using 4,4’-dimethoxyldiphenylacetylene instead of diphenylacetylene, and **3l** was formed in 52% yield. ^1^H NMR (400 MHz, CDCl_3,_ Me_4_Si) δ 3.76 (s, 3H), 3.85 (s, 3H), 6.71 (d, *J* = 8.8 Hz, 2H), 6.88 (s, 1H), 6.90 (d, *J* = 8.8 Hz, 2H), 7.02 (d, *J* = 8.8 Hz, 2H), 7.15 (d, *J* = 8.7 Hz, 2H), 7.37–7.24 (m, 5H); ^13^C NMR (100 MHz, CDCl_3_, Me_4_Si) δ 55.2, 55.3, 113.5, 114.2, 127.3, 127.5, 127.6, 128.2, 130.4, 130.9, 131.7, 132.9, 140.4, 144.1, 158.4, 158.9.

1,1,2,3,4,4-Hexaphenyl-1,3-butadiene (**5**) [62]: To the reaction mixture containing **4a**, CuCl (1 mmol) and TCQ (2 mmol) were added. The reaction mixture was stirred for 12 h at room temperature and compound **5** was isolated in 43% yield. ^1^H NMR (300 MHz, CDCl_3_, Me_4_Si) δ 6.96–7.40 (m, 30H); ^13^C NMR (75 MHz, CDCl_3_, Me_4_Si) δ 127.1, 127.6, 128.0, 128.0, 128.1, 128.2, 139.5, 140.4, 141.1, 142.0.
